# Effects of radiofrequency field from 5G communication on fecal microbiome and metabolome profiles in mice

**DOI:** 10.1038/s41598-024-53842-2

**Published:** 2024-02-12

**Authors:** Xing Wang, Guiqiang Zhou, Jiajin Lin, Tongzhou Qin, Junze Du, Ling Guo, Panpan Lai, Yuntao jing, Zhaowen Zhang, Yan Zhou, Guirong Ding

**Affiliations:** 1 Department of Radiation Protection Medicine, School of Military Preventive Medicine, Air Force Medical University, Xi’an, China; 2 Ministry of Education Key Lab of Hazard Assessment and Control in Special Operational Environment, Xi’an, China; 3 School of Public Health, Shandong Second Medical University, Weifang, China

**Keywords:** Radiofrequency field, Gut microbiota, Metabolite, Mice, Feces, Microbiology, Environmental sciences

## Abstract

With the rapid development of 5G networks, the influence of the radiofrequency field (RF) generated from 5G communication equipment on human health is drawing increasing attention in public. The study aimed at assessing the effects of long-term exposure to 4.9 GHz (one of the working frequencies of 5G communication) RF field on fecal microbiome and metabolome profiles in adult male C57BL/6 mice. The animals were divided into Sham group and radiofrequency group (RF group). For RF group, the mice were whole body exposed to 4.9 GHz RF field for three weeks, 1 h/d, at average power density (PD) of 50 W/m^2^. After RF exposure, the mice fecal samples were collected to detect gut microorganisms and metabolites by 16S rRNA gene sequencing and LC–MS method, respectively. The results showed that intestinal microbial compositions were altered in RF group, as evidenced by reduced microbial diversity and changed microbial community distribution. Metabolomics profiling identified 258 significantly differentially abundant metabolites in RF group, 57 of which can be classified to Kyoto Encyclopedia of Genes and Genomes (KEGG) pathways. Besides, functional correlation analysis showed that changes in gut microbiota genera were significantly correlated with changes in fecal metabolites. In summary, the results suggested that altered gut microbiota and metabolic profile are associated with 4.9 GHz radiofrequency exposure.

## Introduction

In recent decades, information and communication technologies have developed remarkably, numerous electronic communication devices such as mobile telephone and Wi-Fi by using electromagnetic fields (EMF) have become basic necessity of life^1–3^, which has attracted much attention to the influences of electromagnetic fields on human health. Radiofrequency (RF) field is considered to range from 100 to 300 GHz, and has been used for communication purposes as radio waves, especially in mobile phones, base stations, laptops and other wireless communications^4–7^. The 5G networks work in several different frequency bands, with the lower frequencies (below 1 GHz) already used for early mobile communication. Currently, the 5G technology has been expanded to frequencies below the 6 GHz^8,9^. The three main operators that activated their 5G services in China were China Mobile, China Unicom and China Telecom. According to the ministry of industry and information technology of the people’s republic of China, China Mobile (the telecom operator with the largest network and the largest number of customers in China) was allotted the frequency bands at 2.6 GHz and 4.9 GHz, China Telecom and China Unicom were given 2.1 GHz, 3.3 GHz and 3.5 GHz. Furthermore, much higher radiofrequencies (RF), frequencies above 6 GHz, are also used at later stages of technology evolutions.

The introduction of 5G communication devices, which operates in the high frequency part of the electromagnetic spectrum, has drawn more public attention to health concerns. Certain reviews of RF field, at frequencies above 6 GHz, induced biological and health effects have been published^1,8^. Besides, many studies have reported that 0.45–3.8 GHz from phone radiation may have the potential health effects in both animals and humans^7,10–16^. Few biological studies have been done for RF field at frequency 4 GHz-6 GHz^17,18^.

The gut microbiota plays a vital role in human health. Many studies have shown that the gut microbiota influences not only gastrointestinal function but also central nervous system physiology and behavior by regulating the microbiota-gut-brain axis^19–22^. Recently, a few literatures reported the effects of electromagnetic radiation on gut microbiota. Yu et al. found that the Firmicutes/Bacteroides ratio in gut microbes decreased after pulsed electromagnetic field (PEMF) exposure^23^. Luo et al. reported that long-term pulsed electromagnetic field (2450 MHz) exposure not only causes depression-like neurobehavioral disorders, but also leads to an imbalance in the gut microbiota^24^. Currently, we have found that depression-like behavior was induced in mice after 4.9 GHz RF (a commonly used working frequency of 5G communications) exposure^25^.

To date, no public literature has reported the effects of 5G communication frequency exposure on gut microbiome and metabolome profiles. Therefore, this study aimed to explore the effects of exposure to 4.9 GHz RF on fecal microbiome and metabolome profiles, in order to investigate the biological impacts of electromagnetic radiation on the host-gut microbiota interaction.

## Results

### Diversity of gut microbiota

**Figure 1:**
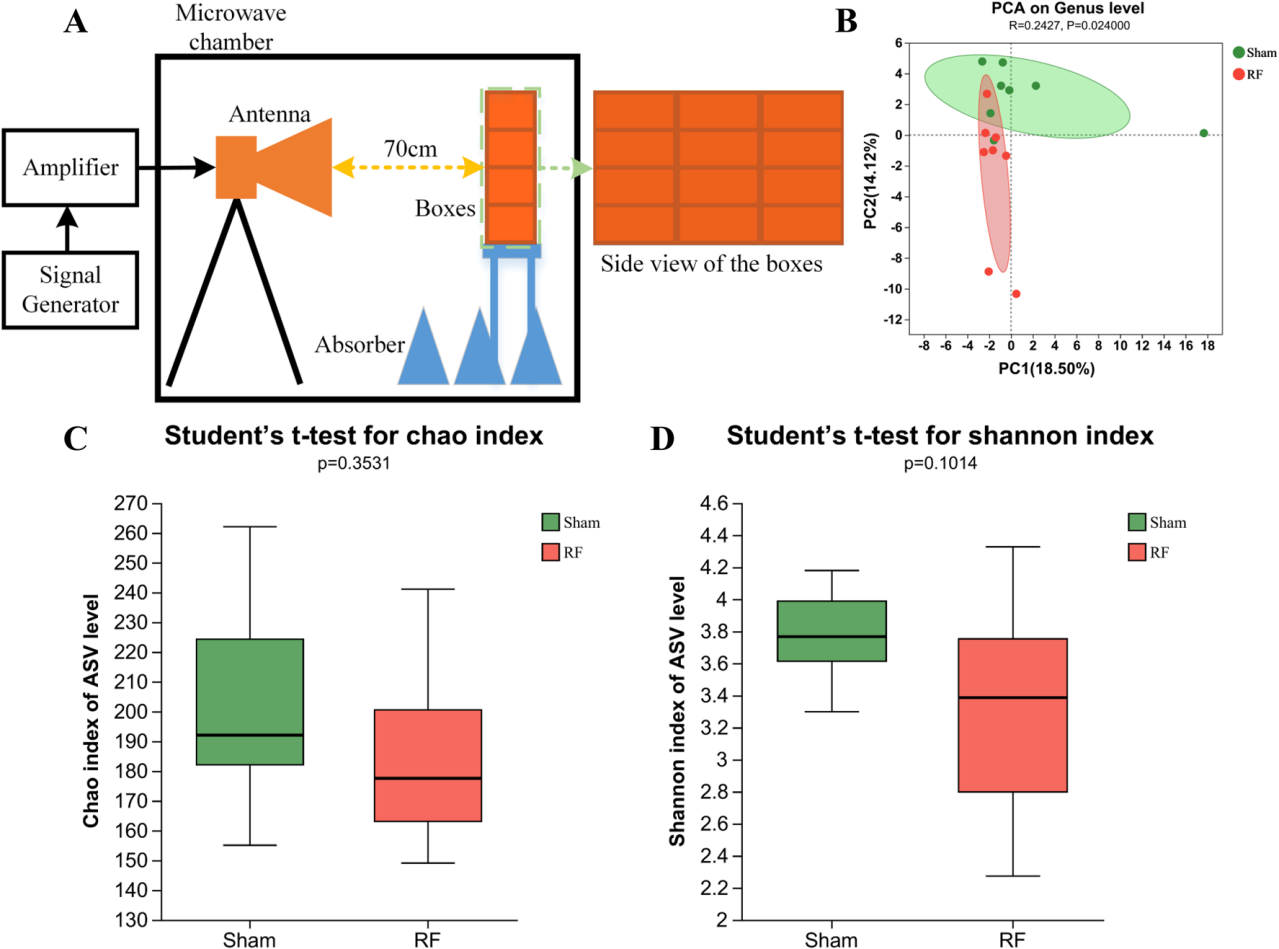
Schematic diagram of experiment set up of the exposure system and the diversity of gut microbial in Sham and RF group. (**A**) Schematic diagram of experiment. (**B**) Principal coordinates analysis (PCA) of the Genus level. (**C**) Chao index of the ASV (Amplicon Sequence Variants) level. (**D**) Shannon index of the ASVlevel.

To clarify the structural diversity of intestinal microbiota in Sham and RF mouse, Chao and Shannon indices of the alpha diversity were used to estimate the richness and diversity, respectively. Compared to the Sham group, a reduction in alpha diversity was observed in RF group, although this disparity did not achieve statistical significance (Fig. 1C,D). In addition, the Principal Component Analysis (PCA) of weighted Bray–Curtis dissimilarity were used to measure beta diversity, which showed that the gut microbial community compositions were different between RF group and Sham group (Fig. 1B).

### Change in gut microbiota composition of RF groups

**Figure 2:**
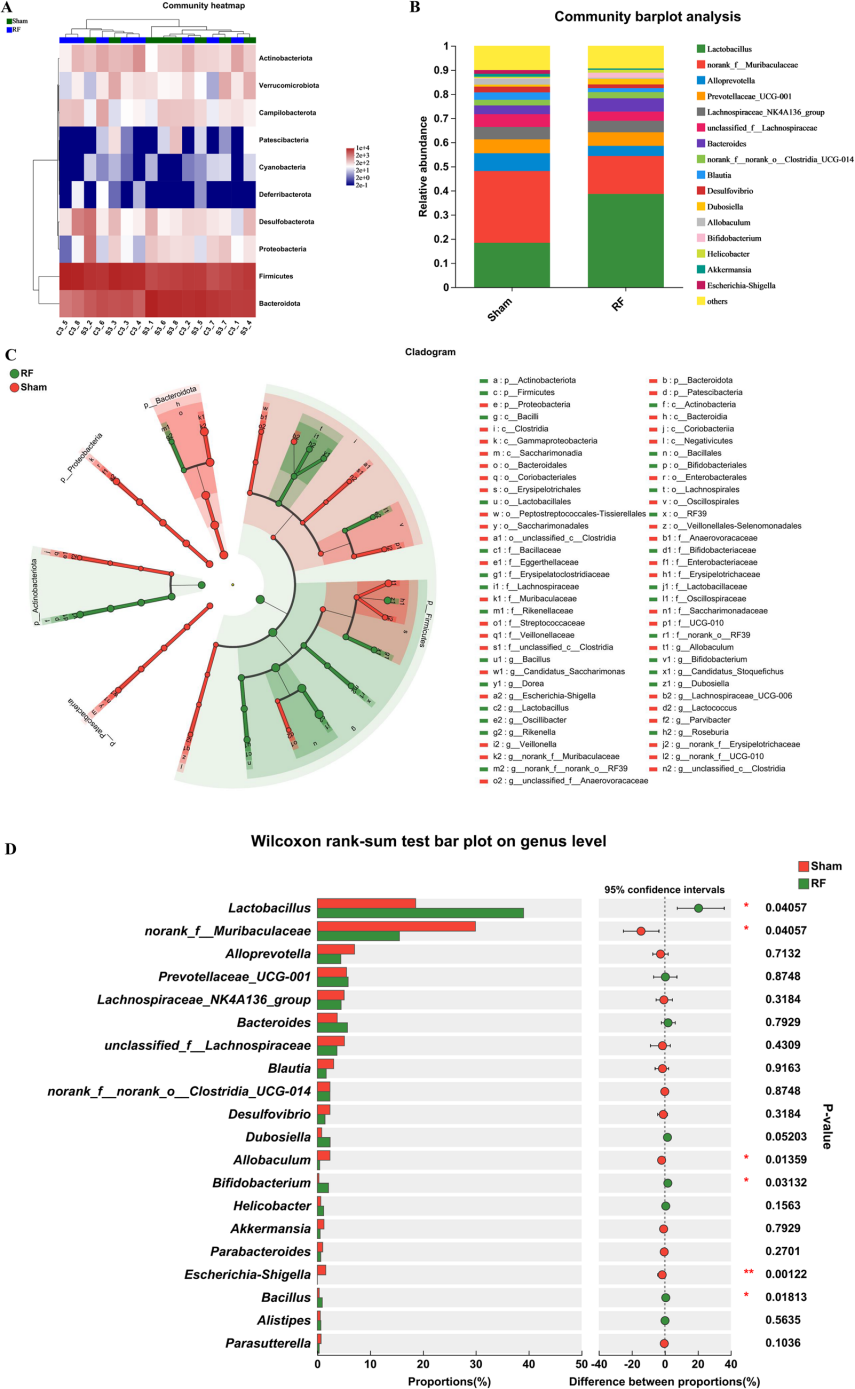
Gut microbiota composition profiles in Sham and RF groups. (**A**) Heatmap analysis of the relative abundances of bacterial phylum between groups sham and RF. (**B**) The relative abundances of microorganisms in Sham group and RF group at genus level. (**C**) LEfSe analysis showed differences from phylum to genus level between Sham group and RF group (LDA score ≥ 2). (**D**) Analysis of microbiome differences at genus level. Only the top 20 genera in relative abundance are shown. Statistical analysis was performed by the Wilcoxon rank-sum test. ^∗∗∗^*P* < 0.001, ^∗∗^*P* < 0.01, and ^∗^*P* < 0.05.

At the phylum level (Fig. 2A), *Firmicutes* and *Bacteroidota* are dominant microbiota in Sham and RF mouse. At the genus level (Fig. 2B), in comparison with Sham group, *Lactobacillus* and *Bacteroides* abundance increased, and *Muribaculaceae* and *Alloprevotella* abundance decreased in the RF group.

LEfSe was used to confirm whether the difference in the enrichment of specific bacteria between RF group and Sham group. Using a LDA score cutoff point 2, in the phyla level, *Bacteroidota* (*P* = 0.046), *Proteobacteria* (*P* = 0.0357) and *Patescibacteria* (*P* = 0.0185) were significantly overrepresented in the feces of Sham group, whereas *Actinobacteriota* (*P* = 0.0157) and *Firmicutes* (*P* = 0.0274) were enriched in RF group. In the Class level, only *Actinobacteria* (*P* = 0.0357) and *Bacilli* (*P* = 0.0274) were enriched in RF group with statistical difference. The cladogram demonstrated the fecal bacterial community structure from phylum to genera, which indicated difference in microbiota phylogenetic distributions between RF and Sham group (Fig. 2C).

Wilcoxon rank-sum tests were used to further compare the difference of fecal microbiota at genus level between the two groups. Of these taxa, the relative expression of *Lactobacillus* (*P* = 0.04057), *Bifidobacterium* (*P* = 0.03132) and *Bacillus* (*P* = 0.01813) in RF group were more than in Sham group with statistically significant, whereas *Escherichia-Shigella* (*P* = 0.00122), *Muribaculaceae* (*P* = 0.04057), and *Allobaculum* (*P* = 0.01359) were significantly enriched in Sham group (Fig. 2D). These results showed fecal flora composition of RF group and Sham group was remarkable different.

### Metabolite identification

Non-targeted metabolomics approach (LC–MS) was performed to examine the fecal metabolites alteration after RF exposure. In total, 1155 metabolites were identified, including pos ion mode and neg ion mode for 504 and 651 respectively, 174 and 201 of which were annotated to KEGG (Table 1). We also investigated the difference in fecal metabolites of mice that result from long-term exposure to 4.9 GHz radiofrequency field, which found 103 and 155 differential metabolites in the positive and negative ion modes, respectively. Compared with the Sham group, 141 metabolites were downregulated while 117 metabolites were upregulated in RF group, and 57 of them could be annotated to KEGG functional pathway (*P* ≤ 0.05, VIP > 1; Table 2).

**Table 1:** Statistics of identified metabolites.

Ion mode	All peaks	Identified metabolites	Metabolites in Library	Metabolites in KEGG
pos	8591	504	408	174
neg	10,249	651	601	201
Total	18,840	1155	1009	375

(1) Ion mode: LC–MS analysis is mainly divided into a positive ion mode and a negative ion mode; (2) All peaks: The number of mass spectra extracted; (3) Identified metabolites: the number of metabolites annotated by level-one MS data, level-two MS data, the search library (self-built, Metlin, HMDB, etc.); (4) Metabolites in library: the number of metabolites assigned to public databases such as HMDB and Lipidmaps; (5) Metabolites in KEGG: The number of metabolites assigned to KEGG (Kyoto Encyclopedia of Genes and Genomes) pathways.

**Table 2:** Statistics of differential metabolites between RF and Sham groups.

Ion mode	RF vs Sham	Downregulated metabolites	Upregulated metabolites
pos	103	65 (15)	38 (7)
neg	155	76 (22)	79 (13)
Total	258	141 (37)	117 (20)

Differential metabolite definition: *p* < 0.05, VIP > 1. The first column was the ion mode. The second column, RF vs Sham, represented the number of differential metabolites in the RF group compared with the Sham group. The third and fourth columns showed the number of differential metabolites with corresponding changes. Outside the brackets were all the number of differential metabolites, and inside the brackets were the number of differential metabolites annotated to the KEGG functional pathway.

### Metabolic variation analysis

**Figure 3:**
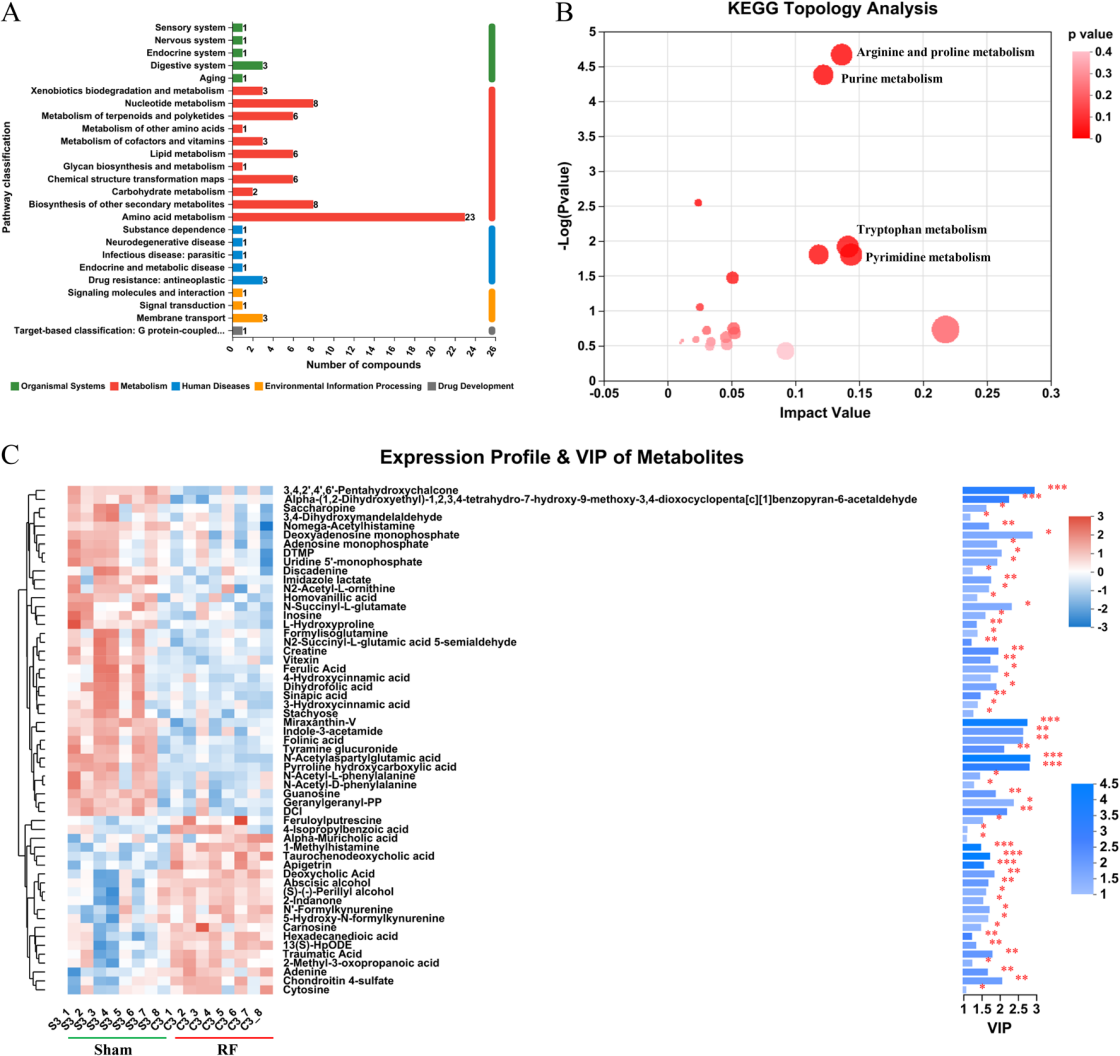
Identification of metabolites. (**A**) KEGG pathway classification: different metabolites detected and annotated. (**B**) KEGG Topology Analysis of differential metabolites. (**C**) Fecal metabolic profiles in Sham and RF group. Red and blue represent the increase and decrease of relative metabolite expression, separately. The histogram in the right is the variable importance in projection (VIP) of metabolites based on the OPLS-DA model. The length of the stripe indicates the contribution of the metabolite to the difference between the two groups, and the greater the value, the greater the difference between the two groups. The color of the stripe indicates the difference of metabolites between the Sham and RF groups, namely the value of *P*_value. ^∗∗∗^*P* < 0.001, ^∗∗^*P* < 0.01, and ^∗^*P* < 0.05.

The different metabolites identified above were assigned to the KEGG databases. All 57 metabolites could be divided into 5 KEGG first-grade pathways, of which 22 and 35 were separately divided into KEGG second-grade pathways under POS and NEG ion modes. The first class of “metabolism” is dominated by “amino acid metabolism”, followed by “nucleotide metabolism”, “biosynthesis of other secondary metabolites”, “lipid metabolism”, “metabolism of terpenoids and polyketides” and “chemical structure transformation maps”. Meanwhile, “Organismal Systems” and “Human Diseases” are the second largest metabolic pathways, and the least metabolites are annotated in “Drug Development” (Fig. 3A). The KEGG Topology analysis showed that differential metabolites were significantly enriched in Arginine and proline metabolism and Tryptophan metabolism and Pyrimidine metabolism (Fig. 3B).

The heat map and VIP of metabolites scores were visualized these 57 significantly different metabolites (Fig. 3C). Overall, 18 and 39 metabolites were separately significantly higher and lower in RF group. Of these, 3,4,2',4',6'-Pentahydroxychalcone, N-Acetylaspartylglutamic acid, Pyrroline hydroxycarboxylic acid and Miraxanthin-V were significantly more abundant in Sham group; Taurochenodeoxycholic acid、1-Methylhistamine and Apigetrin were presented at higher levels with a significant difference in RF group.

### Correlation analysis of gut microbiota and fecal metabolites

**Figure 4:**
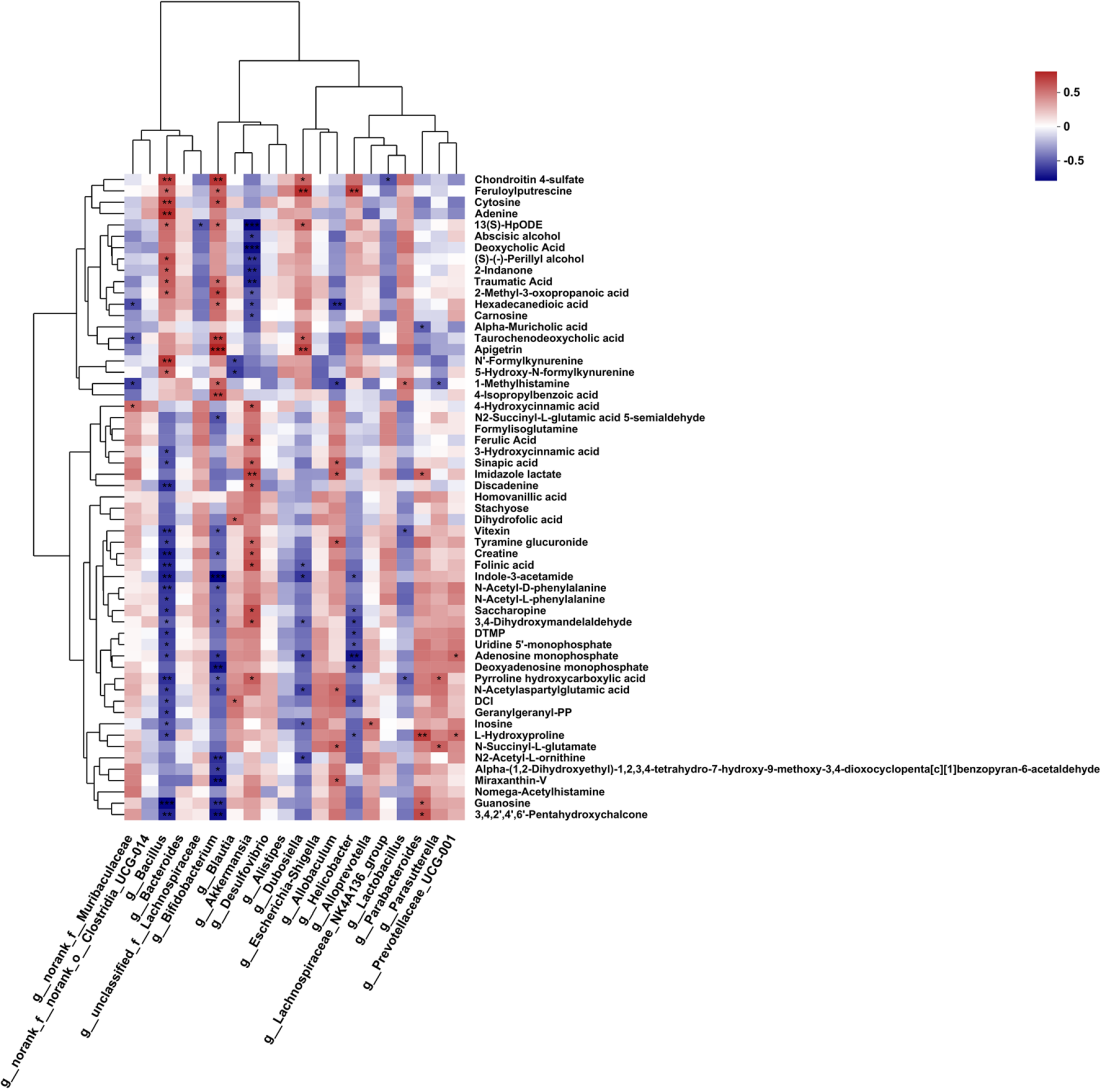
Spearman correlation analysis of the relative abundance of genus level microorganisms and differential metabolites. Positive and negative correlations are respectively displayed in red and blue in the heat map. The strength of the color is proportional to the strength of Spearman correlation. ^∗∗∗^*P* < 0.001, ^∗∗^*P* < 0.01, and ^∗^*P* < 0.05.

Correlation analysis between the altered metabolites noted to KEGG pathway and the relative abundance of the top 20 bacterial genera was calculated using the Spearman’s correlation coefficients. As shown in Fig. 4, *Lactobacillus* and *Muribaculaceae*, the relative abundance of the top 2 genera, showed an opposite correlation with the altered metabolites. *Bacillus* and *Bifidobacterium* were significantly correlated with most metabolites (more than half of the different metabolites). In addition, 1-Methylhistamine was significantly associated with *Lactobacillus* and norank_f_*Muribaculaceae*. Correlation analysis indicated RF group displayed significant difference in the intestinal microorganism, which may lead to a significantly change in the metabolomic profile.

## Discussion

In recent years, the growing utilization of communication devices has led to public concern regarding the potential health hazards associated with RF fields generated by mobile phones. According to the standard released by IEEE International Committee on Electromagnetic Safety in 2019 (C95.1)^26^, the power density exposure of persons permitted in restricted environments at 2–300 GHz spectrum was 50 W/m^2^. Therefore, in this study, 50 W/m^2^ was selected for 4.9 GHz RF field exposure. According to previous studies, EMFs can induce adverse health effects, such as brain tumors, genetic damage, infertility, immune disorders, electromagnetic allergies, leukemia, rapid exhaustibility, increased anxiety, poor learning, and so on^14,27–34^. Increasing demands for greater channel capacity and higher data rates for mobile communication has given rise to exploration of the RF spectrum towards the network system. Ken Karipidis et al. have reviewed 107 experimental studies on the biological and health effects above 6 GHz, which illustrated kinds of biological effects, including cell proliferation and differentiation, genetic virulence, regulation of gene expression or gene control, cell signaling and other effects^1^. Another review reported exposure to RF field below 4 GHz influenced the immune system, gene and protein expression, the neuroendocrine system, and neurobehavior, cerebral blood flow, neural biochemical activity and so on^6^. For 4–6 GHz field, there are few reports. Gang Rui et al. studied the influences of 5.8 GHz microwave radiation on synaptic plasticity in the hippocampus of rats and no positive results were reported.

The role of the microorganisms living the human digestive tract, gut microbiota, on health and disease have been generally studied^35–37^, mainly because they can participate in human metabolism, digestion and absorption, nutrient intake and immune regulation, etc., to maintain normal physiological functions of the host^38^. Moreover, many studies have indicated that intestinal microbiome dysregulation plays a crucial role in the mechanism of radiation-induced intestinal injury^39–41^. While numerous studies have shown the impact of ionizing radiation on the structure of intestinal microbes^42,43^, only a limited number of reports have focused on the effects of electromagnetic radiation^23,24^. The studies on the biological effects of communication frequency band on gut microbiota are still remain inconsistent. Therefore, we used 16S rRNA sequencing to detect changes in enteric microorganism composition after 4.9 GHz radiofrequency exposure.

Gut microorganisms are now known as "host" factors that influence energy intake. The host's immune response and interference may also lead to a decrease in microbial community diversity^44^. Our results showed the gut microbial diversity decreased and the microbial composition structure altered after RF exposure. Similarly, Wang et al. showed a relatively low diversity of microbial flora in larvae exposed to 3.5 GHz RF fields^45^. Yiyi Li et al.^46^ reported a number of *Bacteroidetes*, probiotics in the intestine, can produce short-chain fatty acids such as butyrate to help relieve intestinal inflammation and regulate the integrity of the intestinal mucosal barrier. Meanwhile, Lei Zhong et al.^47^ indicated that the reduced ratio of *Bacteroidetes* to *Firmicutes* (B/F ratio) could partly reflect the intestinal microbial community disorder and intestinal injury in RF process. In our study, the relative abundance of *Firmicutes* markedly increased and the relative abundance of *Bacteroide* decreased without statistical significance (the decreased B/F ratio) in RF group, which suggested that 4.9 GHz radiofrequency radiation may cause intestinal microbiota dysbiosis in mice. Contrary to our results, Yu et al. found that the B/F ratio in gut microbes increased with pulsed electromagnetic field (PEMF) exposure, which has been speculated to be associated with a reduction in obesity^23^. Intriguingly, *Lactobacillus*, the probiotic in *Firmicutes*, showed a significantly increased abundance after 3 weeks RF exposure, which may play a gut-protective role and help improve the gut microbiota. Several studies have already verified lactic acid bacteria can reduce intestinal damage caused by radiation^46,48–50^. Furthermore, the relative abundance of *Muribaculaceae* was significantly reduced in RF group, which was in consistence with the consequence reported by and Luo et al.^24^. Besides, Rachael E. Antwis et al.^51^ investigated the impacts of radiation exposure on the bacterial and fungal microbiome of small mammals in the Chernobyl Exclusion Zone, and found that *Muribaculaceae* was negatively correlated with total absorbed dose rate, therefore, they suggested that *Muribaculaceae* may serve as the biomarker of radiation exposure.

It is widely understood that intestinal microbiota and their metabolites can regulate host metabolism activities, but little is known about their interactions because of variability and complexity^52^. Based on Metabolome profiling, most of the differential metabolites were annotated into amino acid metabolic pathways, including metabolites with top 15 VIP values such as Pyrroline hydroxycarboxylic acid involved in the Arginine and proline metabolism, Indole-3-acetamide (IAM) involved in the Tryptophan metabolism, and N-Acetylaspartylglutamic acid (NAAG) involved in the Alanine, aspartate and glutamate metabolism, with an extremely significant difference. NAAG is a neuropeptide, which has neuroprotective effects by activating metabotropic glutamate receptor 3 (mGluR3) and reducing glutamate release^53^. It was found that lower NAAG level was associated with cognitive deficits in patients with neurodegenerative diseases^54^ and impaired working memory performance in rats^55^. In this study, we found that the level of NAAG significantly reduced in fecal metabolites after RF exposure, which may be associated with RF induced depression-like behavior in mice^25^. Tryptophan is the only precursor of serotonin, a key monoamine neurotransmitter involved in regulating central nervous transmission and physiological function of the gut^56^. Tryptophan can be metabolized to kynurenine, tryptamine, and indole, thereby regulating neuroendocrine and intestinal immune responses. As signaling molecules in tryptophan metabolism, indole and its metabolites play anti-inflammatory and neuroprotective role in activation of aryl hydrocarbon receptor (AHR)^57,58^. Decreased IAM after RF may result in insufficient AHR activation, which may also contribute to RF induced depression-like behavior in mice^25^. Amino acids are participated in biological oxidation, cell metabolism, protein synthesis and other processes to provide energy for the body and the brain^59^. Proteins and amino acids react with free radicals to produce peroxides, which can damage tissues in biological systems, resulting in changes in immune regulation, gastrointestinal function, and body metabolism^60,61^. In this study, the differential metabolites in feces were mainly enriched in arginine and proline metabolism pathways, which may cause damage to the body by affecting protein production. Zhao et al. showed that exposed to multifrequency microwaves of 1.5 GHz and 4.3 GHz produced immune suppressive responses by regulating immune regulation and genes related to cell metabolism in rats^18^. Zhao-lai Dai et al. have put forward that the amino acids-derived molecules produced by gut bacteria could regulate host microbial composition and metabolism^62^.

Accumulating evidence suggests that the gut microbiota may influence neurodevelopment and social behavioral programming in different animal species, and the microbiome-gut-brain axis has received increasing attention. Previous studies have shown that electromagnetic field exposure not only causes depression-like behavior, but also causes intestinal microbiota and serum metabolite disturbance^24^. This is consistent with our previous research that 4.9 GHz RF exposure could induce depression-like behavior, and in this study, it was found that exposure could cause intestinal microbiota disturbance in mice. Xie et al. showed that depression-like behavior is closely related to different bacterial groups in phylum *Firmicutes* and different microbial metabolites, mainly enriched in lipid metabolism and amino acid metabolism^63^, in which tryptophan metabolism plays a key role. Tryptophan metabolism is directly or indirectly regulated by intestinal microorganisms, and its metabolites have immune, metabolic and neuroregulatory functions^64^. At present, a large number of studies have shown that tryptophan metabolism is related to a variety of neurological diseases^64,65^. Luo et al. 's study also suggests that changes in differential metabolites involved in tryptophan metabolism may be a key factor in improving EMF-induced depression-like behavior^24^. In this study, fecal metabolite analysis showed that differential metabolites were significantly enriched in amino acid metabolic pathways, of which tryptophan metabolic was the key pathway. From the correlation networks, *Bacillus* and *Bifidobacterium* were the first two bacteria that are notably correlated with most of the differential metabolites, indicating they contribute greatly to the disturbance of microbial metabolism during RF exposure. However, changes in relative abundance of *Lactobacillus*, the probiotic relevant to anti-inflammation and a variety of metabolic processes^46^, and *Muribaculaceae* are not related with most differential metabolite. To clarify the causal relationship between fluctuations in the intestinal environment caused by RF and the host's situation, in subsequent studies, it is necessary to use the differential metabolites or microorganisms screened in this study to intervene the normal mice, and detect the host's situation such as the level of cognition and emotion in mice.

In conclusion, 4.9 GHz radiofrequency exposure in this research not only caused a significant difference in mouse gut microbiota but also resulted in a metabolic profile change. As of now, we have not obtained direct evidence that metabolic processes were affected by 4.9 GHz RF field. In addition, at present, we only suspect that the gut microbiota and metabolism imbalance were associated with depression-like behavior induced by RF, and metabolic profile imbalance may be related to the alterations of immune regulation or inflammation. Further investigation is needed to reveal the mechanisms of the RF exposure on gut microorganisms and determine the biological significance of the observed phenomena.

## Material and methods

### Animals and experimental design

Nine-week-old male C57BL/6 mice were purchased from the Laboratory Animal Center of the Fourth Military Medical University (Xi’an, China) and raised in the mouse cage (n = 8 for each cage) with a 12-h light and dark cycle. All experimental protocols were approved by the Animal Welfare Committee of the Fourth Military Medical University (IACUC-20210105, Xi’an, China) and strictly based on the ARRIVE guidelines. All mice were adaptation to the laboratory conditions (Temperature, 20–26 °C; Humidity, 45–65%) with adequate food and water. After 1 week of adaptation, the mice were randomly divided into Sham group and RF group (*n* = 8 for each group).

### Study design

The 4.9 GHz RF-exposure system was designed with radiating structure, which was mainly composed of a signal generator (Keysight N5171B, USA), a power amplifier (Bonn BLMA0860-100, Germany), and a double ridged horn antenna (XJT—DR10180, China). Briefly, for the RF group, the mice with conscious were first placed in the plexiglass boxes. The size of each box was 32 mm × 42 mm × 80 mm with small ventilation holes on every side, and the mice were unable to move freely in the box. Then, the boxes with animals (an animal in a box) were laid out right next to each other during exposure as shown in Fig. 1A. The animals were whole body exposed to 4.9 GHz RF field for 21 consecutive days (1 h/day), since in this condition we previously found that 4.9 GHz RF exposure induced depression-like behaviour^25^. During exposure, the mice were awake, but not allowed to access food and water. With the help of plastic boxes, the mice were basically fixed to ensure the defined direction of irradiation. The average power density (PD) was 50 ± 2.5 W/m^2^, which measured in advance by an electromagnetic field meter (PMM8053A, PMM Costruzioni Electtroniche Centro Misure Radio Electriche S.r.l., Milan, Italy). For the Sham group, the animals were processed in the same condition as the RF group, except the exposure control system kept closed. During the exposure protocol, no obvious stress response was found in animals. Fecal samples were collected after the last exposure and placed directly on ice and subsequently stored at −80 °C for microbial and metabolomic analyses.

### Illumina MiSeq sequencing

Microbial DNA was extracted from fecal samples, then DNA purity and concentration were detected by NanoDrop2000. and selected to perform the further sequencing after passing quality inspection by 1% (wt/vol) agarose gels electrophoresis. The V3-V4 region of the bacteria 16S rRNA gene was amplified by PCR (3 min at 95 °C, 27 cycles × 30 s at 95 °C, 30 s at 55 °C, and 45 s at72 °C, and a final extension 10 min at 72 °C, 10 °C until finished) using primers 338F 5′- ACTCCTACGGGAGGCAGCA-3′ and 806R 5′- GGACTACHVGGGTWTCTAAT-3′. The PCR amplification products were identified with 2% (wt/vol) agarose gels, then quantified with a QuantiFluor™-ST UV/Blue Fluorometer Channels (Promega Corporation, USA) and sequenced using Illumina Miseq platform at Majorbio Bio-Pharm Technology Co., Ltd. (Shanghai, China).

### Quantification of metabolites

LC–MS (High Performance Liquid Chromatography-Mass Spectrometry) was used to compare the fecal metabolite signatures of Sham and RF mice. Metabolites were extracted from fecal samples using the following method. 50 mg fecal sample was suspended in 400 μl methanol solution (4:1 methanol:water) with 0.02 mg/mL L-2-chlorophenylalanine for homogenizing by lapping for 6 min (−10 ℃, 50 Hz), and then ultrasonic extraction at low temperature for 30 min (5 ℃, 40 kHz). After 15 min of centrifugation (4 ℃, 13000 g), the resulting supernatant was transferred into clean tubes for analysis. Metabolome profiling was conducted on UHPLC-Q Exactive HF-X platform (Thermo Fisher Scientific, USA) at Majorbio Bio-Pharm Technology Co., Ltd. (Shanghai, China). The mass spectral signals were collected by positive and negative ion scanning modes, respectively. Progenesis QI (Waters Corporation, Milford, USA) was used for peak extraction, alignment and identification of raw data. Finally, a data matrix containing retention time, peak area, mass-to-charge ratio (M/Z) and identification information was obtained for post-processing and bioinformatics analysis.

### Bioinformatics analysis

All bioinformatics analyses were analyzed on the Majorbio Cloud Platform (https://cloud.majorbio.com). The Illumina MiSeq fastq reads were imported into the QIIME2 (quantitative insights into microbial ecology) pipeline, and the optimized data were processed using the sequence denoising method (DADA2). The ASV (amplicon sequence variant), represented the sequence and abundance information, were obtained for further analysis. Alpha diversity was used to evaluate the microbial community diversity, including Chao and Shannon indices (R packages, Version1.6.2). Beta diversity analysis based weighted Bray–Curtis distance was performed to visually evaluate the difference of bacterial communities between Sham and RF groups by principal component analysis (PCA) plots (R packages, Version1.6.2). Statistical differences in microbial relative abundance between Sham and RF mice were identified using the linear discriminant analysis effect size (LEfSe; R packages, Version1.6.2). Wilcoxon rank-sum test was used to test the differences of genus level between the two groups.

For metabolomic analysis, the raw data of LC/MS is preprocessed by Progenesis QI (Waters Corporation, Milford, USA) software and then uploaded to the Majorbio cloud platform (https://cloud.majorbio.com) for data analysis. Student’s t-test was used to compare metabolite profiles between two groups. In order to clarify the physic-chemical and biological functional properties of metabolites, the online KEGG (Kyoto Encyclopedia of Genes and Genomes, http://www.genome.jp/kegg) databases was used for level-one and level-two identification and annotation. The importance of each metabolite was ranked according to the variable importance in projection (VIP) scores calculated by the orthogonal partial least-squares discrimination analysis (OPLS-DA) model using Python (Version1.0.0). Spearman correlation analysis was used to assess the correlation between fecal metabolites and intestinal microbiota (Python, Version1.0.0).

### Ethical approval

Animal experiments were performed following guidelines from the Animal Welfare Committee of the Fourth Military Medical University (IACUC-20210105, Xi’an, China) and strictly followed the ARRIVE guidelines.

## Data Availability

The datasets presented in this study can be found in online repositories. The names of the repositories and accession numbers can be found below: SRA, NCBI: PRJNA799826; MetaboLights: MTBLS4186.
